# Parenting styles and personal belief in a just world among Chinese children and adolescents: gender, living location, and age as moderators

**DOI:** 10.3389/fpsyg.2024.1357667

**Published:** 2024-07-04

**Authors:** Jing Wang, Yonghong Ye, Yun Wang, Xihua Zeng

**Affiliations:** ^1^Department of Psychology, School of Public Health, Southern Medical University, Guangzhou, China; ^2^Department of Psychiatry, Zhujiang Hospital, Southern Medical University, Guangzhou, China; ^3^State Key Laboratory of Cognitive Neuroscience and Learning, Faculty of Psychology, Beijing Normal University, Beijing, China

**Keywords:** parenting styles, personal belief in a just world, Chinese children and adolescents, age, living location

## Abstract

**Introduction:**

The positive development of the personal belief in a just world (PBJW) plays a vital role in academic achievement and mental health among children and adolescents. Therefore, it is necessary to understand the influencing factors of PBJW better. The association between parenting styles and PBJW has been well established, but whether this association varies among different groups remains an open issue. The present study aimed to examine the strength of the associations between parenting styles and PBJW among Chinese children and adolescents and the role of certain moderators (gender, living location, and age) in these associations.

**Methods:**

This study employed hierarchical regression and simple slopes analyses to examine data from the National Children's Study of China. The database includes 24,013 Chinese children and adolescents in grades 49 (*M* = 12.76 years, *SD* = 1.73), with 53.50% boys.

**Results:**

The results indicated that (1) authoritative parenting was positively correlated with PBJW; (2) both authoritarian and permissive parenting styles were negatively correlated with PBJW; (3) the positive relationship between authoritative parenting and PBJW was more prominent in urban regions; the negative relationship between authoritarian and PBJW was stronger in urban regions; and the negative relationship between permissive parenting and PBJW was more pronounced among girls and older children and adolescents.

**Discussion:**

These findings highlight important associations between parenting styles and the development of PBJW among Chinese children and adolescents, and suggest strategies for policy-makers, educators, and parents to improve PBJW for different types of Chinese children and adolescents.

## 1 Introduction

The personal belief in a just world (PBJW) refers to people who believe they live in a fair world where they can get what they deserve (Lerner and Miller, 1978). PBJW is a crucial resource for the development of children and adolescents. It provides them with a sense of safety and control by making them believe they are not at the mercy of random disasters, thus motivating them to strive for a better future (Hafer and Rubel, 2015; Chen et al., 2022). Children and adolescence are critical periods for the development of PBJW. Therefore, exploring the antecedents of PBJW development during this time is essential for fostering its growth. The family plays a crucial role in the development of children and adolescents (Bronfenbrenner, 1986; Repetti et al., 2002). Previous research has indicated that parenting styles, as a core element of the family, have a strong positive relationship with the development of PBJW in children and adolescents (Dalbert and Sallay, 2004). However, these studies have primarily focused on Western cultural contexts. Existing research shows significant differences in parenting styles across different cultural backgrounds, not only in cross-national and regional comparisons (Taris and Semin, 1998; Kremers et al., 2003) but also among different age and gender groups within the same culture. In China, where a collectivist culture predominates (Diener and Diener, 1995; Leung et al., 2004), the role of the family is particularly important. However, there is a lack of research examining the relationship between parenting styles and PBJW within the Chinese cultural context, as well as the potential moderating effects of gender, living location, and age on this relationship (Liu et al., 2019; Quan, 2021). This study aims to explore the relationship between parenting styles (authoritative, authoritarian, and permissive) and PBJW, and to identify differences in this relationship among Chinese children and adolescents of different genders, living locations, and ages. The answers to these questions can help expand our understanding of the factors influencing PBJW and assist policymakers, educators, and parents in taking effective measures to enhance PBJW based on the characteristics of children and adolescents.

### 1.1 The relationship between parenting styles and PBJW

Parenting styles are a combination of parenting attitudes, behaviors and their impact on children's emotional behavior (Darling and Steinberg, 1993). Maccoby and Martin (1983) and Baumrind (1991) proposed a typology of parenting styles, classifying them into three types based on two underlying processes: responsiveness and demandingness. Responsiveness refers to the degree of attention and support that parents provide to their children, while demandingness refers to the degree of regulation and supervision that parents impose on their children (Baumrind, 1991). This typology includes three parenting styles: authoritative, authoritarian, and permissive (Baumrind, 1971).

Authoritative parents are both highly demanding and highly responsive. They guide their children's behavior through clear and democratic rules and actively respond to their needs (Baumrind, 1991). Authoritarian parents are highly demanding but low in responsiveness. They expect absolute obedience from their children, characterized by strict control and a lack of warmth and support (Baumrind, 1971, 1991). Permissive parenting is characterized by low levels of demandingness. Although permissive parents may be diverse in responsiveness (with either permissive-neglectful or permissive-indulgent styles), they are tolerant and accept children's misbehavior with little punishment or restriction (Baumrind, 1971; Alizadeh et al., 2011). The lack of parental control may also cause children's externalizing behaviors (Alizadeh et al., 2011). Different parenting styles differ in their ability to meet children's needs for autonomy, relationship, and competence (Ryan and Deci, 2017; Abidin et al., 2022; Wei et al., 2022), which can directly affect children's perceptions of whether they believe their lives are fair and in turn affect their PBJW development (Resh and Sabbagh, 2017).

Numerous Western studies have shown that authoritative parenting is considered the optimal parenting style, being more effective in promoting various aspects of children's successful development, such as better academic performance, higher maturity and development levels, and fewer behavioral problems (Lamborn et al., 1991; Masud et al., 2015). This is because authoritative parenting is characterized by positive interaction and responsiveness to children's needs, providing discipline and clear boundaries (Baumrind, 1971). In contrast, Western research has shown that authoritarian and permissive parenting styles have a negative impact on children's development in areas such as emotional intelligence and mental health (Baumrind, 1971). This is because authoritarian parenting, due to its lack of responsiveness to the child, and permissive parenting, due to its over-indulgence of the child, leaves the child without clear boundaries in their behavior (Barton and Hirsch, 2016; Shaw and Starr, 2019). However, some Western studies have noted that authoritarian parenting shows certain benefits for African Americans (Deater-Deckard et al., 1996) and Hispanic Americans (Pinquart and Kauser, 2018).

Particularly regarding the optimal parenting style for Chinese children and adolescents, research findings have revealed inconsistent empirical results. While some studies support that authoritative parenting is beneficial for the successful development of Chinese children and adolescents (Huang and Prochner, 2003), some found that the positive correlation between the optimal parenting style (i.e., authoritative) and academic achievement was stronger or clearer among European Americans but weaker or unclear among Asians (Dornbusch et al., 1987). Moreover, compared to their Caucasian counterparts, Asian high school students reported higher levels of authoritarian parenting from their parents (Dornbusch et al., 1987), which is characterized by strictness, control, and high involvement in children's lives (Chao, 1994). This parenting style does not appear to have a negative impact on the development of Asian students and is even associated with positive outcomes, such as higher academic self-efficacy and lower depression (Li et al., 2010).

Research findings indicate that the effectiveness of parenting styles in fostering the development of children and adolescents varies, which can be explained by the cultural background of parenting, as families evolve within societal systems characterized by specific cultural values and beliefs (Palacios et al., 2022). Thus, although parents may be the same, the degree to which children feel loved, valued, and connected to their families can vary significantly depending on the cultural background (Chen et al., 2024). In Chinese culture, the term “authoritarian” has different connotations (Chao, 1994). Influenced by traditional Confucian values, Chinese society is characterized by collectivism, social harmony, and respect for elders and authority (Markus and Kitayama, 1991). In this culture, the roles and responsibilities of family members are strictly defined, with each member expected to adhere to their role, thereby maintaining overall harmony and order (Chao, 1994). Consequently, in Western cultures, control or strictness is sometimes equated with domination, manifesting as parental hostility, aggression, mistrust, and dominance (Dornbusch et al., 1987). In contrast, in China, parental control, along with children's obedience and loyalty, primarily aims to ensure social harmony and family integrity (Chao, 1994). Consequently, such authority- and discipline-based parent-child relationships are generally accepted by Chinese children, who often perceive them as expressions of parental care, love, or involvement rather than as unfairness (Lamborn et al., 1991; Chao, 2001).

Much of the current research in China focuses on academic achievement and does not include PBJW as an outcome variable. Additionally, China has undergone significant social, cultural, and economic changes (Li, 2020), including urbanization and rural-urban migration trends (Wang et al., 2024). Therefore, the relationship between parenting styles and PBJW remains unclear in China, and further clarification is needed to determine whether cultural differences regarding optimal parenting styles also affect the relationship between parenting styles and PBJW.

The development of PBJW is fundamentally based on the establishment of trust (Dalbert and Sallay, 2004). Authoritative parenting provides a harmonious family atmosphere with consistent and fair rules, aiding children's emotional development and fostering a sense of security, trust, and fairness, which enhances their confidence in the predictability and fairness of life (Booth, 1994; Dalbert and Sallay, 2004). China is a collectivist country that emphasizes interpersonal relationships. The development of fairness and trust is especially important for children's PBJW because it helps them generalize this trust to believe that the world and the people around them are fair to them. Therefore, we posit that in the Chinese context, authoritative parents who meet children's and adolescents' expectations for fair treatment (such as warmth, autonomy, and competence) are positively correlated with PBJW.

Authoritarian parents focus on authority and order, expect their children to accept their ideas and judgments without reservation, and have a high degree of control over their children's behavior, but rarely respond positively to their children's needs (Baumrind, 1971; Baumrind et al., 2010). Although authoritarian parenting emphasizes consistency in rules, which helps children predict future injustices, the lack of respect for children's perspectives in rule-making can cause internal conflict (Baumrind, 1971). This parenting style may lead to children and adolescents being more likely to develop suspicious and apathetic personalities, as well as having more difficulty believing that life is fair (Radziszewska et al., 1996; Liu and Merritt, 2018). In addition, children and adolescents have difficulty obtaining the treatment they expect from their parents, such as access to autonomy and caring (Baumrind, 1991), which makes them more likely to assess their lives as unjust (Umemura and Šerek, 2016; Hofer and Spengler, 2018). Therefore, authoritarian parenting may impair the development of PBJW in children and adolescents. However, whether this holds true in the Chinese cultural context remains unclear. As mentioned earlier, authoritarian parenting is not necessarily negative in the Chinese context. Additionally, Chinese children with different characteristics may have varying degrees of understanding of authoritarianism and control (Lin and Wang, 2022). Hence, it is necessary to further clarify the relationship between authoritarian parenting and PBJW among Chinese children with different characteristics.

Permissive parenting is characterized by low demands. They generally have low expectations for their children and provide minimal monitoring of their behavior. This lack of clear and consistent rules can make it difficult for children to perceive a sense of security and fairness in their environment (Baumrind et al., 2010; Barton and Hirsch, 2016). As justice is closely linked to rules, the lack of coherent rules makes children uncertain about whether they will be victims of an unforeseen fate (Dalbert and Sallay, 2004). In addition, these parents fail to provide adequate support and guidance for the development of children and adolescents (Milevsky et al., 2007; Barton and Hirsch, 2016), leaving children and adolescents with more unfair experiences. Thus, permissive parenting is negatively associated with PBJW.

In summary, the present study aimed to fill this research gap by exploring the relationship between parenting styles and PBJW and its differences in different groups of people. The present study aims to fill the gap in existing research by exploring the relationship between parenting styles and PBJW and its variations across different groups to further understand the key factors influencing the development of PBJW in children and adolescents. Our study not only highlights the positive role of authoritative parenting styles in providing a harmonious and predictable family environment, but also reveals the possible negative impact of authoritarian and permissive parenting styles on PBJW development. In addition, the present study takes into account differences in parental expectations among children and adolescents in different cultural and environmental contexts, which helps to explain differences in the effects of parenting styles across cultures and environments.

### 1.2 Variations in the relationship between parenting styles and PBJW among Chinese children and adolescents of different genders, living locations and ages

Whether the relationship between parenting styles and PBJW varies across Chinese children and adolescents with different characteristics remains an open question. Previous research has noted that the effects of parenting styles vary by gender, age, and living location (Uji et al., 2014; Huang et al., 2024). Despite having similar parenting styles, children's perceptions of love, being valued, and fairness may differ depending on the specific context (Baumrind, 1971). However, fewer studies have examined the effects of parenting styles across these different characteristics in the context of Chinese culture. Jasso (1990) proposed that individuals' evaluations of justice depend not only on the actual rewards they receive from others but also on the rewards they expect based on their personal needs, social rules, or comparisons with others. Empirical studies point out that the same experience of justice can lead to different perceptions of fairness among individuals with different intensities of need for fair treatment (Resh, 2010; Resh and Sabbagh, 2017). Children and adolescents with different characteristics (e.g., gender, living location and age) may have different needs for fair treatment (Resh, 2010). Thus, gender, living location and age may interact with parenting styles to influence PBJW development, but there is still little research on this moderating effect.

There are several reasons to examine gender-specific pathways from parenting styles to children and adolescents' PBJW. First, studies have pointed out differences in the neural mechanisms of moral sensitivity between women and men (Harenski et al., 2008). Specifically, women tend to adopt care-based moral evaluations, and men tend to adopt justice-based moral evaluations (Harenski et al., 2008). Because there is a clear difference in the need for warmth and clear rules between girls and boys (Eagly et al., 2000; Shek, 2002), this can affect how they respond to different parenting styles. Second, China is dominated by Confucianism, with its emphasis on the maintenance of a patriarchal culture, which has led to significant gender role differences (Chao, 1994; Leung et al., 2004). Because boys are encouraged from an early age to be strong and responsible, and they will desire more independence and freedom from their parents (Hou et al., 2020). The research indicates that boys are more inclined to resist authoritarian parenting (Yang et al., 2018). In contrast, girls are taught from an early age to be more obedient and submissive and maintain relationships, and they desire more warm treatment from their parents (Bi et al., 2018; Zhu et al., 2021). In addition, girls are more susceptible to emotional messages regarding relationships and social support than boys (Cyranowski et al., 2000; Lewis et al., 2015), and they place greater value on positive relationships with their parents (Yao et al., 2022). Baumrind (1971) showed that in African American families, authoritarian parenting fosters greater confidence in girls but not in boys. These studies suggest that different parenting styles may have different effects on boys and girls, and these effects may vary across different cultures. However, current research often overlooks whether the impact of different parenting styles on PBJW also varies by gender. In the context of Chinese culture, where significant gender differences exist (Hofstede, 2001), the gender differences in the relationship between parenting styles and PBJW may be more pronounced. Therefore, further research is needed to explore how different parenting styles influence the development of PBJW in boys and girls in the Chinese context.

Living location may moderate the relationship between parenting styles and children and adolescents' PBJW. With the acceleration of reform and opening up and globalization, the influence of Western liberal ideology is stronger in cities (Chen and Li, 2012; Lin and Wang, 2022). Because cities have a stronger democratic and liberal atmosphere, children and adolescents growing up in such environments have a stronger sense of freedom and independence and perceive parent-child relationships as more equal (Lin and Wang, 2022). As a result, they are more eager for their parents to treat them in an autonomous manner (Lin and Wang, 2022). In contrast, in rural areas, due to limitations such as economic and social living conditions, which give children and adolescents a narrower range of social contacts, this leads to a lesser influence of their Western liberal ideas (Chen and Li, 2012). As a result, rural areas have a stronger culture of collectivism, patriarchal authority and filial piety, and children and adolescents are taught early to be submissive, respect authority and maintain family relationships (Chen and Li, 2012; Wang et al., 2024). This leads to rural children and adolescents have a weaker need for autonomy (Guo et al., 2005). Previous studies have indicated that among Chinese urban adolescents, perceived authoritarian parenting styles tend to diminish their love and gratitude toward their parents over time, whereas this trend is not observed among rural adolescents (Lin and Wang, 2022). In summary, parenting styles have different effects on the development of children and adolescents in urban and rural areas. However, there is currently no research comparing the differences in the relationship between different parenting styles and PBJW among urban and rural children and adolescents.

Previous research has shown that the relationship between parenting styles and children's developmental outcomes varies by age (Suárez-Relinque et al., 2019). A meta-analysis indicates that the relationship between parenting styles and subjective well-being may vary across different age groups (Huang et al., 2024). These remind us that the relationship between parenting styles and PBJW may exhibit different patterns across various ages. This may be because children of different ages have different needs for parenting styles. Specifically, as children grow older, they face challenges in exploring new learning environments, coping with physiological changes, and establishing their identities. At this stage, they require more parental love, moderate control, and guidance (Soenens et al., 2015; Lo et al., 2020). For instance, research suggests that as children age, neglecting parenting styles can have more significant negative impacts (Lo et al., 2020). Furthermore, the older children become, the more urgently they need to develop a sense of adulthood, and the more they desire their parents to respect their ideas and communicate with them as equals (Steinberg and Silverberg, 1986; Peterson et al., 2005; De-Juanas et al., 2020). Equality and mutual respect play an increasingly important role in developing children's sense of justice (Piaget, 2013). In summary, if parents fail to adjust their parenting styles according to the psychological needs of children and adolescents at different ages, it may affect their PBJW. Specifically, authoritarian parenting styles, which disregard children's autonomy, may be more challenging for older children and adolescents to accept. Therefore, further research is needed to explore how different parenting styles influence the development of PBJW in children and adolescents across different age groups.

Although a few studies have discussed the link between parenting styles and PBJW, most have focused on a single dimension of parenting styles. In addition, due to the different needs for fair treatment among children and adolescents with different characteristics (e.g., gender, living locations and age), this reminds us to further clarify that the relationship between different parenting and children and adolescents' PBJW varies across groups.

### 1.3 Hypotheses of the current study

**Figure 1:**
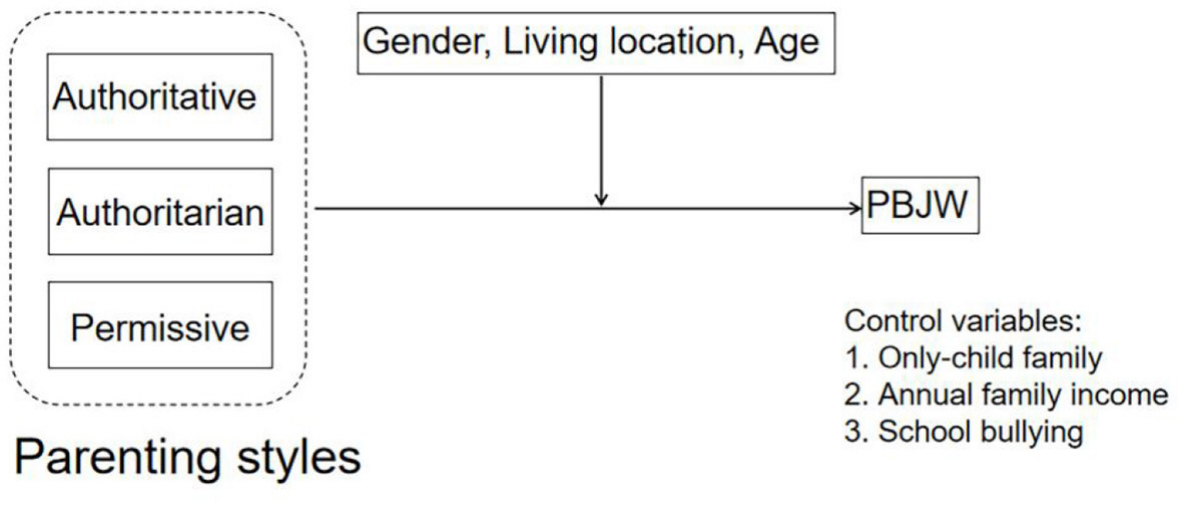
A model of the association between parenting styles and PBJW across different genders, living locations and ages. These data control for the only-child family, annual household income and school bullying.

The purpose of this study is to explore the relationship between different parenting styles and PBJW in children and adolescents and to test the potential moderating effects of gender, living location, and age on this relationship. Specifically, the objectives are: (1) to identify the potential impact of different parenting styles on PBJW in children and adolescents. Specifically, authoritative parenting is positively associated with PBJW, and authoritarian and permissive parenting styles are negatively associated with PBJW. (2) to examine whether demographic variables such as gender, living location, and age act as moderating variables that influence the relationship between different parenting styles and PBJW. Based on the preceding discussion, the following hypotheses are proposed, as shown in Figure 1.

## 2 Methods and measures

### 2.1 Participants

The data for this study were derived from the Social Adaptation Database of the National Children's Study of China (NCSC). The NCSC aims to assess the performance of Chinese children and adolescents aged 6-15 years in terms of their academic, cognitive and psychosocial development (Dong and Lin, 2011). Developed by the State Key Laboratory of Cognitive Neuroscience and Learning at Beijing Normal University, the database is China's first large-scale basic database on the psychological development of children and adolescents covering national, regional, and urban/rural areas. To ensure that the sample is representative and random at all stages, the NCSC sample was selected from 100 counties in 31 provinces in China using a multi-stage, stratified, and unequal probability sampling method. More details about the design of the NCSC can be found in its general report (Dong and Lin, 2011). The social adjustment database used in this study covered data from 24,013 children and adolescents in grades 4-9 (12.76 ± 1.73), of whom 53.50% (12,839) were boys and 46.50% (11,174) were girls. 61.18% (14,690) of the children and adolescents were from rural areas, whereas 38.82% (9,323) were from urban areas. For specific information (see Table 1). Consent was obtained from the parents of the participants and the headmaster of the school prior to the study.

**Table 1:** Distribution of the number of subjects with respect to grade (age), gender, living location and only children (*N* = 24,013).

**Grade**		**Grade 4**	**Grade 5**	**Grade 6**	**Grade 7**	**Grade 8**	**Grade 9**
Gender	Boys	2,184	2,164	2,175	2,184	2,068	2,064
	Girls	1,819	1,848	1,833	1,821	1,934	1,919
Living location	Urban	1,524	1,528	1,525	1,555	1,606	1,585
	Rural	2,479	2,484	2,483	2,450	2,396	23,980
Only-child	Yes	1,850	1,700	1,719	1,611	1,580	1,571
	No	2,153	2,312	2,289	2,394	2,422	2,412
Age (*M ± SD*)		10.42 ± 0.86	11.43 ± 0.83	12.48 ± 0.84	13.50 ± 0.78	14.33 ± 0.80	14.42 ± 1.21

### 2.2 Measures

#### 2.2.1 Personal belief in a just world

*The PBJW Scale* was developed by Dalbert (2002). The scale is a 7-item self-assessment scale used to assess the status of children's PBJW (e.g., “In general, things about me in life are just”). Participants were required to respond on a 4-point Likert scale with answers ranging from 1 (*strongly disagree*) to 4 (*strongly agree*) (Dong and Lin, 2011). The higher the scale score, the higher the children's PBJW. The scale was originally a 6-point scale, but considering that the sample included children in the fourth grade, their level of psychological and cognitive development could not meticulously understand and differentiate the intensity of the PBJW. Therefore, the PBJW scale used by the National Children's Study of China (NCSC) was a 4-point scale to improve the accuracy of the measurement. Cronbach's alpha coefficient of this scale in the present study was 0.80.

#### 2.2.2 Parenting styles

*The Parenting Styles Scale* was used to evaluate children and adolescents' perceptions of parenting behavior (Robinson et al., 1995). This scale is a self-assessment scale including 34 items, which features three subscales for authoritative (16 items), authoritarian (12 items) and permissive parenting styles (6 items). Authoritative parenting consists of three dimensions: warmth (7 items), democratic participation (4 items) and rationality (5 items). Authoritarian parenting was measured in terms of three dimensions: physical punishment (5 items), irrationality (4 items) and verbal aggression (3 items). The permissive parenting included 6 items. The scale is a five-point scale (1 = *never*, 5 = *always*). The higher the mean score on each subscale, the stronger the children's experience of a particular parenting. In this study, Cronbach's alpha coefficients for the subscales of authoritative, authoritarian and permissive parenting styles were 0.92, 0.89 and 0.71, respectively.

#### 2.2.3 Living location

Living location was reported by principals, reflecting whether their school was located in an urban area or a rural area. By the Compulsory Education Law of the People's Republic of China (Standing Committee of the National People's Congress, 2006), children and adolescents should attend school nearby so the school location is equivalent to the living location. This variable was divided into rural and urban areas, with rural areas coded as 0 and urban areas coded as 1.

### 2.3 Control variables

Annual household income and being an only child affect the resources children can obtain, impacting children and adolescents' PBJW (Liu and Jiang, 2021; Quan, 2021). In addition, the school experiences of children and adolescents (school bullying) play an important role in the development of PBJW (Peter and Dalbert, 2010). Therefore, these three variables were selected as control variables in this study. The annual household income is yuan, as the basic monetary unit in China, was coded from 1 (*less than 3,000 RMB, equal to* ~*453 U.S. dollars*) to 9 (*200,001 RMB or more, equal to* ~*30,199 U.S. dollars*). This variable was reported by parents and reflected the total actual income of all family members per year, which was converted into an ordinal scale. Only-child families are distinguished according to the number of siblings in the child's family (0 = *not the only-child family*, 1 = *only-child family*). For this study, 42.60% of families were only-child families. *The School Bullying Scale* was assessed using the Chinese version of *the Olweus (**1993**) school bullying* questionnaire (Zhang and Wu, 1999; Dong and Lin, 2011). The scale is a single dimension (bully dimension) with seven items (e.g., “I was teased or made fun of”). Each child responded from 1 (*no*) to 5 (*more than 5 times*) depending on his/her situation. The Cronbach's alpha coefficient for the scale in this study was 0.78.

### 2.4 Data analysis

Before performing the hierarchical multiple regression analyses, we first filled in the missing data using SPSS 26.0. For categorical variables, we used the mode fill method. For continuous variables, we began by performing Little's MCAR test and found that the *p*-value was less than 0.001. This result indicated that the study data did not conform to the assumption of Missing Completely At Random (MCAR). To further understand the pattern of missing data, we conducted a missing data pattern analysis and found a correlation between missing data and observations, suggesting that our data may be consistent with Missing At Random (MAR). In addition, the maximum percentage of missing data does not exceed 7 %. Based on these findings, we processed the missing data through the Expectation Maximization (EM) method using SPSS, setting the number of EM iterations to 25. In addition, this study used the Harman's one-factor test (Podsakoff et al., 2003), which revealed the presence of 8 factors with eigenvalues greater than 1. The first factor accounted for 22.37% of the variance, which is below the 40% threshold criterion (Wingate et al., 2018), indicating that our study was not significantly affected by common method bias. Lastly, before performing regression analysis, we employed the Variance Inflation Factor (VIF) method to check for multicollinearity among variables. The VIF values were all below 10, confirming the absence of significant multicollinearity issues.

## 3 Results

### 3.1 Correlations among parenting styles, gender, living location, age and PBJW

Pearson correlation analysis was performed to identify the relationships among continuous variables. Point biserial correlation analysis was conducted to examine the associations between dichotomous variables and continuous variables (see Table 2). Spearman correlation analysis was used to assess the correlations between ordinal variables and other variables and the correlations among binary variables. The correlation analysis showed that the study variables were correlated in the expected directions. In this context, authoritative parenting was positively and significantly correlated with PBJW, while authoritarian and permissive parenting styles were negatively and significantly correlated with PBJW.

**Table 2:** Descriptive statistics and correlation analyses among variables (*N* = 24,013).

**Variables**	**1**	**2**	**3**	**4**	**5**	**6**	**7**	**8**	**9**	**10**
Only-child family	1									
Annual household income	0.23^***^	1								
School bullying	−0.06^***^	−0.07^***^	1							
Gender	0.09^***^	0.01^*^	0.07^***^	1						
Living location	0.31^***^	0.28^***^	−0.07^***^	−0.01	1					
Age	−0.09^***^	−0.08^***^	−0.06^***^	0.00	−0.02^**^	1				
Authoritative	0.16^***^	0.12^***^	−0.19^***^	0.04^***^	0.12^***^	−0.10^***^	1			
Authoritarian	−0.04^***^	−0.04^***^	0.28^***^	0.08^***^	−0.02^**^	−0.03^***^	−0.38^***^	1		
Permissive	−0.10^***^	−0.10^***^	0.15^***^	0.10^***^	−0.13^***^	−0.04^***^	−0.12^***^	0.28^***^	1	
PBJW	0.10^***^	0.08^***^	−0.23^***^	−0.01	0.10^***^	−0.13^***^	0.40^***^	−0.20^***^	−0.10^***^	1
*M*	0.42	3.58	0.73	0.53	0.39	12.76	2.19	3.29	1.69	2.96
*SD*	0.49	1.94	0.73	0.5	0.49	1.73	0.79	0.84	0.63	0.54

N = 24,013. PBJW, personal belief in a just world. Point biserial correlation coefficients are shown for only-child family, gender and living location. Only-child family: 0 = not the only-child family; 1 = only child family. Gender: 0 = girls; 1 = boys. Living location: 0 = rural; 1 = urban. Spearman correlation coefficients are shown for annual family income. ^*^p < 0.05, ^**^p < 0.01, ^***^p < 0.00.

### 3.2 Testing the moderating roles played by gender, living location and age

This study used a hierarchical regression model featuring PBJW as the dependent variable and the only-child family, annual household income and school bullying as control variables to assess the moderating roles played by gender, living location and age in the relationship between parenting styles and PBJW. To avoid multicollinearity, we first standardized annual household income, school bullying, age and parenting styles prior to the regression and included only-child family, gender, and living location as dummy variables (Aiken and West, 1991). We further generated interaction terms for gender, living location and age with the three different parenting styles. In the first step, we added only-child family, annual household income, and school bullying as control variables. In the second step, gender, living location, age and three parenting styles were added. In the third step, we added interactions of predictor and moderator variables. To explain the interaction effects, we analyzed simple slopes and plotted significant interaction effects using both high and low levels of moderating factors (mean ± one standard deviation) (Aiken and West, 1991).

The results showed that after controlling for only-child family, annual household income and school bullying, authoritative parenting was positively correlated with PBJW, but authoritarian and permissive parenting styles had negative correlations with PBJW, explaining a total of 20.00% of the total variance (see Table 3).

**Table 3:** Hierarchical multiple regression analysis of the moderating roles of gender, living location and age (*N* = 24,013).

**Predictor variable**		**Model 1**			**Model 2**			**Model 3**		
		β	**Bootstrap 95%CI**		β	**Bootstrap 95%CI**		β	**Bootstrap 95%CI**	
			**LL**	**UL**		**LL**	**UL**		**LL**	**UL**
	(Constant)		2.920	2.938		2.937	2.959		2.932	2.954
Step 1	Only-child family	0.075^***^	0.069	0.097	0.008	−0.004	0.023	0.008	−0.004	0.023
	Annual household income	0.050^***^	0.020	0.034	0.005	−0.004	0.009	0.005	−0.004	0.009
	School bullying	−0.220^***^	−0.126	−0.113	−0.152^***^	−0.089	−0.076	−0.152^***^	−0.089	−0.076
Step 2	Gender				−0.008	−0.022	0.003	−0.008	−0.021	0.004
	Living location				0.039^***^	0.030	0.057	0.038^***^	0.029	0.056
	Age				−0.109^***^	−0.066	−0.053	−0.107^***^	−0.065	−0.052
	Authoritative				0.337^***^	0.177	0.190	0.310^***^	0.158	0.180
	Authoritarian				−0.036^***^	−0.027	−0.013	−0.023^*^	−0.024	−0.001
	Permissive				−0.027^***^	−0.021	−0.008	−0.044^***^	−0.035	−0.013
Step 3	Authoritative × Gender							0.006	−0.009	0.018
	Authoritative × Living location							0.029^**^	0.010	0.037
	Authoritative × Age							−0.007	−0.011	0.003
	Authoritarian × Gender							0.000	−0.014	0.014
	Authoritarian × Living location							−0.023^**^	−0.033	−0.005
	Authoritarian × Age							−0.010	−0.012	0.002
	Permissive × Gender							0.027^**^	0.007	0.033
	Permissive × Living location							−0.013	−0.026	0.001
	Permissive × Age							−0.020^**^	−0.017	−0.004
	*F*	525.167^***^			667.486^***^			338.174^***^		
	*R^2^*	0.061			0.200			0.202		
	*ΔF*	525.167^***^			693.223^***^			7.228^***^		
	*ΔR^2^*	0.061			0.139			0.002		

*N* = 24,013. PBJW, personal belief in a just world. Only-child family: 0 = not the only-child family; 1 = only-child family. Gender: 0 = girls; 1 = boys. Living location: 0 = rural; 1 = urban. The β*s* were standardized regression coefficients. ^*^p < 0.05, ^**^p < 0.01, ^***^p < 0.001. CI, confidence interval; LL, lower limit; UL, upper limit.

**Figure 2:**
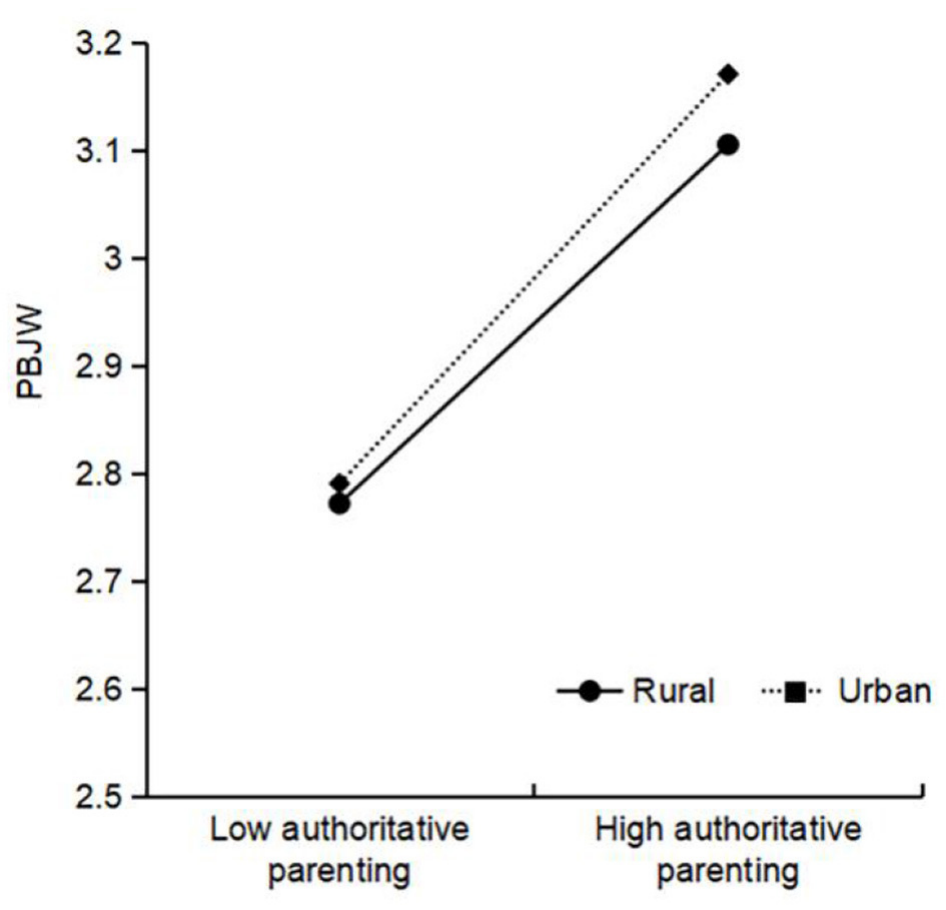
The interactive effect of authoritative parenting and living location on the PBJW. PBJW, Personal belief in a just world.

**Figure 3:**
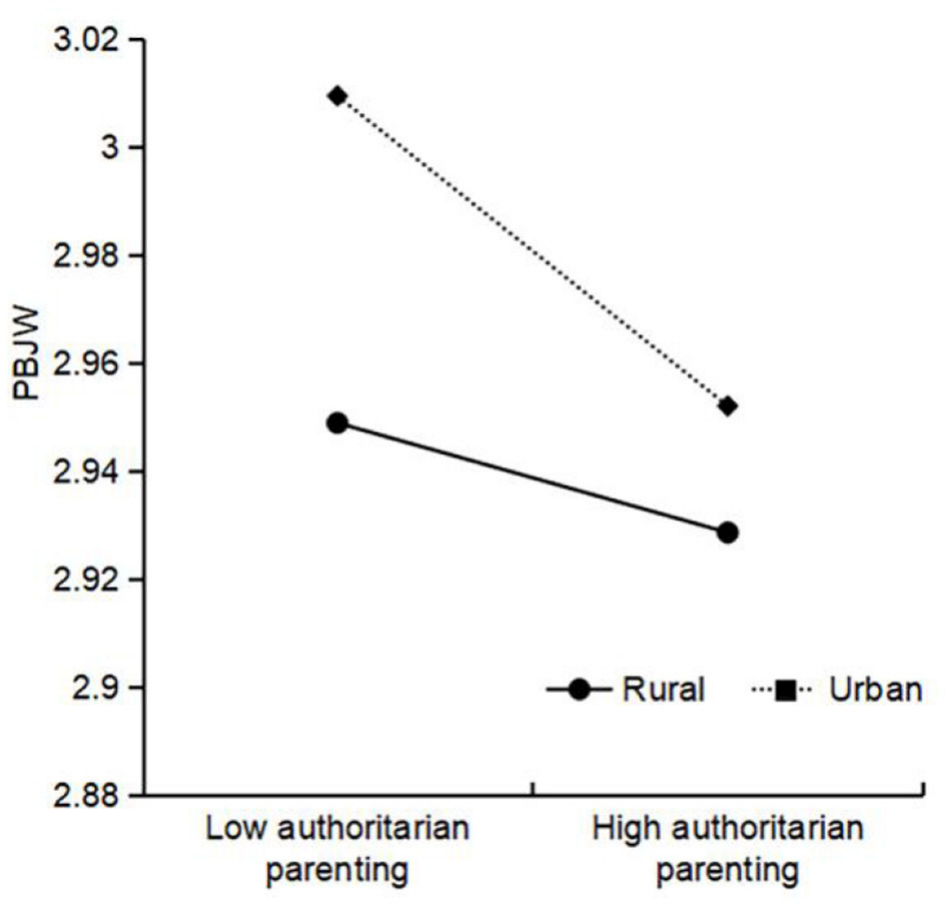
The interactive effect of authoritarian parenting and living location on the PBJW. PBJW, Personal belief in a just world.

**Figure 4:**
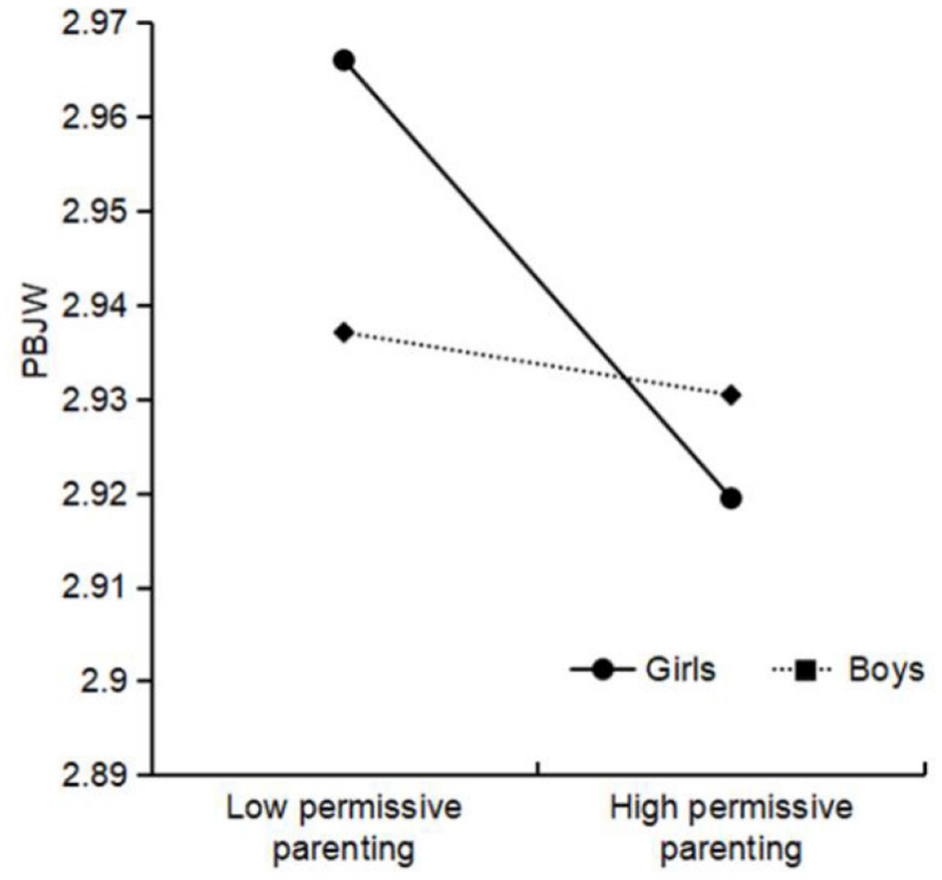
The interactive effect of permissive parenting and gender on the PBJW. PBJW, Personal belief in a just world.

**Figure 5:**
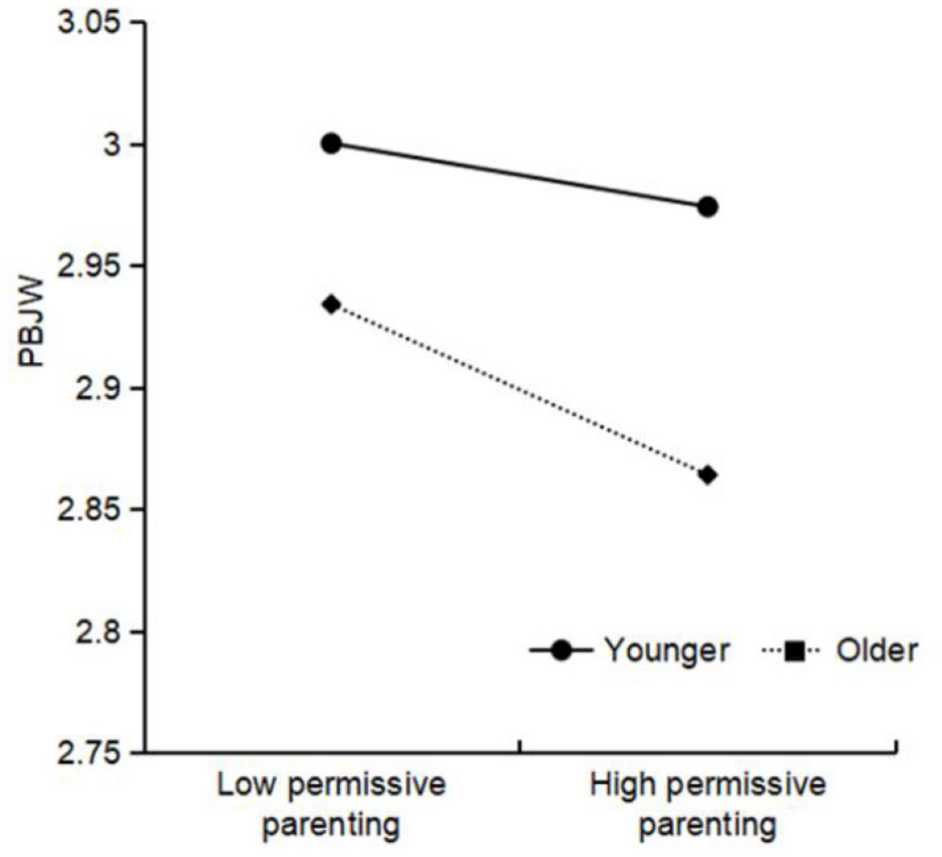
The interactive effect of permissive parenting and age on the PBJW. PBJW, Personal belief in a just world.

Authoritative parenting × living location was significant for PBJW, and further simple slope analysis (see Figure 2) showed that the positive relationship between authoritative parenting and PBJW was stronger among urban areas (β_simple_ = 0.19, *t* = 32.24^***^) than among rural areas (β_simple_ = 0.17, *t* = 27.42^***^). Authoritarian parenting × living location was significant for PBJW, and the simple slope results (see Figure 3) indicated that the negative relationship between authoritarian parenting and PBJW was stronger among urban areas (β_simple_ = −0.03, *t* = −4.67^***^) than among rural areas (β_simple_ = −0.01, *t* = −1.59). Permissive parenting × gender was significant for PBJW, and the simple slope results (see Figure 4) indicated that the negative relationship between permissive parenting and PBJW was stronger in girls (β_simple_ = −0.02, *t* = −4.33^***^) than in boys (β_simple_ = −0.00, *t* = −0.64). Permissive parenting × age was significant for PBJW, and the simple slope results (see Figure 5) indicated that the negative relationship between permissive parenting and PBJW was stronger in older children and adolescents (β_simple_ = −0.04, *t* = −5.32^***^) than in younger children and adolescents (β_simple_ = −0.01, *t* = −2.08^*^).

Due to the small coefficients on the interaction effects and the large sample size, we conducted effect size checks in the follow-up study. The results showed that the interaction effect of authoritative parenting style with living location showed a large effect size among urban children and adolescents (Cohen's d = 0.691) and a medium effect size among rural children and adolescents (Cohen's d = 0.500). The interaction effect of authoritarian parenting style with living location showed a medium effect size for urban children and adolescents (Cohen's d = 0.431) and a smaller effect size for rural children and adolescents (Cohen's d = 0.228). The interaction of permissive parenting styles with gender produced a small effect size in the boys group (Cohen's d = 0.192) and a slightly larger effect size in the girls group (Cohen's d = 0.224). In addition, the interaction effect of permissive parenting style with age was a small effect size in older children and adolescents (Cohen's d = 0.228) and a small effect size in younger children and adolescents (Cohen's d = 0.187).

## 4 Discussion

PBJW is an important psychological resource for children and adolescents and plays a key role in their positive development. However, there is a relative paucity of research on the relationship between parenting styles and PBJW, particularly in terms of considering differences in this relationship across cultural contexts. Secondly, there are also differences in how children and adolescents understand and respond to different parenting styles within different groups within the same culture. In addition, previous studies have focussed on a single parenting style, but few studies have covered the three main parenting styles. This study emphasizes that the investigation of the sense of justice needs to take full account of individual differences in different expectations of fair treatment (Resh and Sabbagh, 2017). Children and adolescents of different genders, living locations and ages may have different expectations of fair treatment, and the links between parenting styles and PBJW may differ among these groups. Therefore, we used the social development database of the National Children's Study of China (NCSC) to examine changes in the relationship between parenting styles and PBJW across gender, living location, and age. We found that authoritative parenting was positively associated with PBJW and that this relationship was more significant in urban areas. Authoritarian parenting was negatively associated with PBJW, and this association was stronger in urban areas. Permissive parenting was negatively associated with PBJW, and this association was stronger in girls, older children and adolescents.

### 4.1 The relationship between parenting styles and PBJW

We found that authoritative parenting was positively associated with PBJW, whereas the other two were negatively associated with PBJW. The relationship between authoritative and authoritarian parenting styles and children's adjustment has been the subject of much debate in families from Chinese backgrounds (Zhang et al., 2017), and these findings provide evidence to address this question. These findings are consistent with previous findings (Dalbert and Sallay, 2004; Umemura and Šerek, 2016; Hofer and Spengler, 2018), further providing generalizable evidence for these relationships. Authoritative parents are characterized by clear rules, warm emotional support and clear communication (Baumrind, 1971; Lavrič and Naterer, 2020). This type of parenting promotes the development of trust in children and adolescents and fulfills their expectations of fair treatment, which leads to positive feelings about justice (Dalbert and Sallay, 2004). Previous studies suggest that authoritarian parenting has less adverse effects on children and adolescents in China than in Western countries (Chao, 2001; Xie et al., 2016; Tang et al., 2018). This is due to the strong atmosphere of collectivism that prevails in China, which emphasizes conformity, social cohesion, and harmony. In such a social environment, children and adolescents may perceive the behavior of authoritarian parents as supportive and caring, rather than as a restriction or violation of rights (Chen et al., 2012; Han et al., 2023). Our results show that authoritarian parenting is negatively associated with PBJW in children and adolescents. This is because in today's China, children and adolescents' self-awareness is gradually awakening, and they gradually disapprove of authoritarian parenting, which negatively impacts the development of PBJW (Zhang et al., 2017; Chen et al., 2024). Permissive parenting does not identify children and adolescents' real needs in time and provides consistent standards for their behavior (Baumrind et al., 2010), which is detrimental to their PBJW development. However, in this study, due to the limitations of the measurement, permissive parenting was defined broadly to include both parenting styles that allow children to be neglected and allowed to develop freely, and parenting styles that are over-indulgent but managed leniently and punished inconsistently. This broad definition suggests that we need to be more careful in interpreting the effects of permissive parenting and consider its specific implications in practical applications. In particular, a recent study shows that warm but not strict permissive parenting is positively associated with Chinese children's academic self-concepts (Chen et al., 2024). In summary, the current findings suggest that authoritative parenting styles (i.e., parenting that combine warmth with appropriate control) have the best performance for PBJW development in children and adolescents.

### 4.2 Gender as a moderator between parenting styles and PBJW

We found that the positive correlation between authoritative parenting and PBJW did not differ between boys and girls. For both boys and girls, authoritative parenting featuring warmth and moderate control fully meets children and adolescents' expectations (Kaufmann et al., 2000) and facilitates the positive development of their PBJW. This finding further emphasizes the importance of authoritative parenting in shaping children and adolescents' good development (Masud et al., 2019; Ali et al., 2023). We did not find differences in the negative relationship between authoritarian parenting and PBJW across genders. This result may be attributed to the fact that, in the context of China's deepening reform and opening up, girls' awareness of their rights has gradually increased, and their quest for individual autonomy has expanded. As a result, both boys and girls are unable to accept authoritarian parenting. The negative association between permissive parenting and PBJW was stronger in girls than boys. Girls are more concerned about the involvement and warmth of others, whereas boys seek more independence and freedom (Perez, 2012; Zhu et al., 2021). Our findings are consistent with previous studies showing that lack of parental support and weak parental relationships are greater risk factors for girls (Lewis et al., 2015; Liu, 2021). This may indicate that permissive parenting has a more negative impact on girls than boys in the Chinese cultural context, as this parenting style has no mature expectations for children and adolescents, while lacking effective emotional support, which makes girls more likely to feel indifferent and unfair. These results suggest that parents should be aware of gender differences in the needs that children and adolescents exhibit during parenting, such as girls' greater need for interpersonal and emotional support.

### 4.3 Living location as a moderator between parenting styles and PBJW

The positive association between authoritative parenting and PBJW was more pronounced in urban areas than in rural areas, which does not suggest that authoritative parenting is not important for rural students. The fact may be that other factors may mask the impact of authoritative parenting on the PBJW of rural children and adolescents. These factors could include a lack of material resources, lower levels of parental education, and less advanced educational philosophies (Chen and Li, 2012; Lin and Wang, 2022). This finding reminds us that further research is still necessary to explore these possibilities.

The negative association between authoritarian parenting and children and adolescents' PBJW was stronger in urban regions compared to rural regions. This is because children and adolescents are more likely to show higher levels of acceptance of authoritarian parenting style in areas where collectivism and traditional culture are strong (Chen and Li, 2012; Yim, 2022; Ali et al., 2023).

The strength of the negative association between permissive parenting and children and adolescents' PBJW did not differ significantly between urban and rural areas. This is because permissive parenting tends to manage children and adolescents too loosely, with a lack of consistency in the rules set and punishments enforced (Xie et al., 2016; Ali et al., 2023). The children and adolescents in this study were in grades 4 to 9, a critical stage for developing a sense of rules and justice (Dalbert and Sallay, 2004), during which parental guidance is essential (Lo et al., 2020). Therefore the negative effects of this parenting style did not differ between urban and rural areas.

### 4.4 Age as a moderator of the relationship between parenting styles and PBJW

There was no significant difference in the relationship between authoritarian parenting styles, authoritative parenting styles and PBJW among Chinese children and adolescents of different ages. This finding may be because children and adolescents in grades 4-9 typically crave autonomy and warmth (Tian et al., 2014). Authoritative parenting adequately meets the desired treatments for children and adolescents of different ages, such as the need for consistent rules, independence, and warmth. In contrast, authoritarian parents tend to arbitrarily use hostile control or harsh punishment to gain compliance, rarely providing explanations or allowing verbal concessions (Baumrind, 1971, 2013), thus failing to satisfy the needs of children and adolescents for warmth and autonomy. The negative association between permissive parenting styles and PBJW was more pronounced among older children and adolescents. This result may be due to the fact that the mean age of children and adolescents in this study was about 12.76 years. In the moderated analysis, the age of children and adolescents in the higher age group should be 14.49 years. At this stage, they need to deal with physical and academic changes (Yu et al., 2013). Permissive parenting reflects lower control over children and adolescents and disengagement from parental responsibilities (Patock-Peckham and Morgan-Lopez, 2006; Baumrind, 2013). In China, permissive parenting is more often seen as a sign of parental irresponsibility (Chen et al., 2000; Lo et al., 2020). This pattern of behavior may lead older children and adolescents to interpret it as a lack of concern and indifference. This attitude may affect their ability to build trust and a sense of security (Chen et al., 2000; Umemura and Šerek, 2016), and may also lead to difficulties in adapting to the normative demands of schooling (Barton and Hirsch, 2016; Xie et al., 2016), resulting in lower PBJW.

### 4.5 Implications

This study is one of only a few studies to explore the relationship between parenting styles and PBJW based on children and adolescents' characteristics in the Chinese context. It has certain theoretical and practical implications. In terms of theory, our study contributes to the literature concerning the development of PBJW by drawing attention to how different parenting styles are associated with Chinese children and adolescents' PBJW. Additionally, we explored the moderating roles of gender, living location and age in the relationship between parenting styles and Chinese children and adolescents' PBJW, thereby supporting the need for examinations of PBJW development to consider the different characteristics of children and adolescents since they will have different treatment expectations. From a practical perspective, this study provides a strategic reference for parents to promote the development of their children and adolescents' PBJW. This study suggests that parents should try to use authoritative parenting that balances warmth and control in daily parenting and be cautious about using negative parenting styles. In addition, parents should be more sensitive to their children and adolescents' needs and rights and adjust their parenting methods to their children and adolescents' realities.

### 4.6 Limitation

While our findings have important implications, this study also faces certain limitations. First, this study collected data from children and adolescents via self-reports, which may lead to difficulty reflecting the true state of parenting styles, especially as children and adolescents may underestimate the quality of the interactions between parents and children, especially in the case of older children who wish to prove their independence. Second, this study employs a cross-sectional design, and so no causal relationships can be inferred. Third, the results obtained regarding a sample population from an Eastern, collectivistic culture are not necessarily applicable to the West and should be replicated in the context of other Western countries to test their generalizability. Additionally, it is worth noting that PBJW is just one dimension of an individual's belief in a just world (BJW); the other dimension is general belief in a just world (GBJW). Considering that the GBJW is more closely associated with abstract and macro-level factors, this study did not cover GBJW. However, distinguishing between these two dimensions remains critical. Although Western studies show mixed results regarding the impact of GBJW on individual development (including both positive and negative effects), most studies in China have found that GBJW has a positive impact on individual development. Therefore, investigating the influencing factors of GBJW among Chinese children and adolescents is meaningful. Ideally, future research should explore the relationships and differences between family factors and both PBJW and GBJW. Finally, it is worth pointing out that the coefficients on the interaction effects remain relatively small despite the large sample size of this study. Therefore, in order to verify the robustness of these results, we recommend more similar studies in the future.

## Data availability statement

The original contributions presented in the study are included in the article/supplementary material, further inquiries can be directed to the corresponding author.

## Ethics statement

Ethical review and approval was not required for this study in accordance with the local legislation and institutional requirements. The studies were conducted in accordance with the local legislation and institutional requirements. Written informed consent for participation in this study was provided by the participants' legal guardians/next of kin.

## Author contributions

JW: Conceptualization, Data curation, Methodology, Software, Validation, Writing – original draft, Writing – review & editing. YY: Conceptualization, Writing – original draft. YW: Conceptualization, Data curation, Writing – review & editing. XZ: Conceptualization, Funding acquisition, Resources, Supervision, Validation, Writing – review & editing.
